# Risk Factors and Innovations in Risk Assessment for Melanoma, Basal Cell Carcinoma, and Squamous Cell Carcinoma

**DOI:** 10.3390/cancers16051016

**Published:** 2024-02-29

**Authors:** K. Wunderlich, M. Suppa, S. Gandini, J. Lipski, J. M. White, V. Del Marmol

**Affiliations:** 1 Department of Dermatology, Hôpital Erasme, Université Libre de Bruxelles, 1070 Brussels, Belgium; katharina.wunderlich@hubruxelles.be (K.W.); judith.lipski@ulb.be (J.L.);; 2 Department of Dermatology, Institute Jules Bordet, Université Libre de Bruxelles, 1070 Brussels, Belgium; 3 Molecular and Pharmaco-Epidemiology Unit, Department of Experimental Oncology, European Institute of Oncology, IRCCS, 20139 Milan, Italy

**Keywords:** skin cancer risk factors, melanoma, basal cell carcinoma, squamous cell carcinoma, risk scores

## Abstract

**Simple Summary:**

Skin cancer is a common, preventable condition of global importance. Different types of skin cancer, like melanoma, basal cell carcinoma, and squamous cell carcinoma, have various risk factors, including UV exposure and genetics. Identifying these risk factors is crucial for targeting early detection and nuanced treatment. Recent research has developed risk scores to identify individuals at high risk, enhancing prevention efforts. This advancement is significant as it improves our ability to detect and treat skin cancer early, ultimately reducing its impact on society.

**Abstract:**

Skin cancer is the most frequently diagnosed cancer globally and is preventable. Various risk factors contribute to different types of skin cancer, including melanoma, basal cell carcinoma, and squamous cell carcinoma. These risk factors encompass both extrinsic, such as UV exposure and behavioral components, and intrinsic factors, especially involving genetic predisposition. However, the specific risk factors vary among the skin cancer types, highlighting the importance of precise knowledge to facilitate appropriate early diagnosis and treatment for at-risk individuals. Better understanding of the individual risk factors has led to the development of risk scores, allowing the identification of individuals at particularly high risk. These advances contribute to improved prevention strategies, emphasizing the commitment to mitigating the impact of skin cancer.

## 1. Introduction

Skin cancer is the world’s most common malignancy in populations with skin types I–III according to the Fitzpatrick classification. The incidence of melanoma and non-melanoma skin cancer (NMSC) has been increasing in recent decades and will persist in its upward trend. The World Health Organization (WHO) estimates the incidence of melanoma and NMSC at over 1.7 million new cases worldwide for 2025 [1]. Prolonged exposure to ultraviolet (UV) radiation is the primary risk factor, making skin cancer largely preventable through lifestyle modifications [2,3]. However, there is a need for further research to better understand and define the individual risk factors associated with skin cancer, to identify individuals at risk and provide early detection and therapy. Public health strategies recommend full body skin examinations for early diagnosis and to reduce skin cancer burden, but evidence supporting visual skin examination’s efficacy in the general population is lacking [4]. So, how can skin cancer screening be made more effective and tailored to individual needs? For this purpose, there are various approaches integrating environmental, genetic, and behavioral factors, to develop risk scores for precise identification of high-risk individuals who require more detailed examination or tailored follow-ups [5,6,7]. However, before establishing risk scores for the identification of high-risk patients, it is essential to look at known risk factors and the latest scientific findings. Only through a well-founded understanding of the underlying pathomechanisms will it be possible to develop risk assessment tools.

The objective of this review is to provide a comprehensive overview of the current state of skin cancer research, focusing on known risk factors for the three commonest types of skin cancer (melanoma, BCC and SCC) and innovations in individuals risk assessment. The aim is to establish a solid foundation for the ongoing and increasingly important discussion on risk scores and preventive measures. A thorough literature search was conducted across multiple bibliographic databases, with particular attention to recent publications. Data searches were specifically conducted on Medline (National Library of Medicine, 8600 Rockville Pike, Bethesda, MD 20894, USA) using the PubMed interrogation interface. Only English language articles were considered.

## 2. Risk Factors for Melanoma

Many risk factors for the development of cutaneous melanoma have been identified by epidemiologic studies. These can be divided into extrinsic (environmental, behavioral) factors (Table 1) and intrinsic (genetic) factors (Table 2).

### 2.1. Extrinsic Risk Factors for Melanoma

Sun exposure is broadly accepted as the key environmental driver of cutaneous melanoma [8]. In 1992, the International Agency for Research on Cancer (IARC) concluded that the whole spectrum of UV radiation was carcinogenic to humans [8]. The main source of UV radiation exposure is the sun (solar radiation). Estimating the cases of melanoma that are attributable to UV radiation exposure is challenging, as solar UV radiation is ubiquitous and the carcinogenic effects are dependent on latitude and the individual’s behavior [9,10]. In Canada, an estimated 62.3% of all melanomas can be linked to an increase in UV radiation exposure, comparing the incidence in 2015 with a 1920 cohort. Similar results are available for Australia, where approximately 63.3% of melanomas can be attributed to exposure to solar UV radiation [11,12]. However, the individual sun exposure pattern seems to be crucial. Specifically, intermittent exposure during activities like sunbathing, water sports, and vacations in sunny places plays a causative role in the development of melanoma, with a documented 60% elevated risk (relative risk (RR): 1.61, 95% confidence interval (CI): 1.31–1.99). Chronic cumulative sun exposure (e.g. occupational exposure) is not associated with melanoma risk in a metanalysis (RR: 0.95, 95% CI: 0.87–1.04) [9]. This observation could be explained by epithelial thickening through enhanced keratinocyte proliferation in response to (chronic) sun exposure, which has been shown to be UV-protective, independent of pigmentation [13]. Intermittent intense sun exposure, on the other hand, stimulates melanocytes: a single erythemal UVB exposure causes a delayed, dose-dependent increase of melanocyte proliferation, whereas the same amount of fractionated suberythemal UVB radiation has no discernible effects. Consequently, nevi and melanoma might be most effectively induced by intermittent UVB overexposure [14].

Sunburns are often used as markers for intermittent sun exposure, as they indicate a lack of adaptation in untanned skin. A history of sunburn is proven to be a significant risk factor for cutaneous melanoma, with a RR of 2.03 (95% CI: 1.73–2.37) [9]. Retrospective epidemiological data suggest that the susceptibility to the carcinogenic effects of solar radiation for melanoma is particularly pronounced during childhood, especially intermittent sun exposure during this period [15]. Experiments in mouse models demonstrate that a single dose of burning UV radiation to neonates, but not adults, is necessary and sufficient to induce tumors resembling human melanoma with high penetrance [16]. Sunburns in childhood, therefore, represent a particular risk for the development of cutaneous melanoma [17].

Intentional UV exposure, whether from the sun or artificial sources, represents another notable risk factor for melanoma. ‘Ever’ intentional sun exposure is linked to an elevated melanoma risk of 1.44 (95% CI: 1.18–1.76) [11]. Notable distinctions in intentional tanning were observed across diverse latitude regions: European countries exhibited markedly higher levels of intentional sun exposure compared with their non-European counterparts. Additionally, pairwise comparisons indicate that intentional tanning is performed at a significantly higher frequency in Sweden and Italy than by individuals in other countries. Conversely, individuals in the UK and Poland show a significantly lower frequency of intentional tanning than the broader European sample. Overall, women express a stronger desire for a deeper tan and report more frequent intentional tanning [18]. For Canada, it was estimated that 18% of melanomas in 2015 could be attributed to sunbathing [11]. Similar results were calculated for Italy, where approximately 13% of melanomas in the year 2008 were attributable to high intentional sun exposure [19].

Not only is exposure to natural UV radiation relevant, artificial UV sources carry a risk too: the ‘ever’ use of tanning beds is associated with an approximately 20% increased risk of melanoma (RR: 1.2, 95% CI: 1.08–1.34). This risk rises by 1.8% (95% CI: 0–3.8%) with each additional session of sunbed use per year, indicating a dose-response effect. Commencing tanning at a very early age increases the risk even further: individuals with initial use of sunbeds before the age of 35 face a 59% higher risk of developing cutaneous melanoma (RR: 1.59, 95% CI: 1.36–1.85). The burden resulting from sunbed use can be quantified with specific figures: in Western European countries, approximately 5.4% of melanomas in the population are estimated to be attributable to the use of tanning beds. For women, the percentage is even as high as 6.9% of all melanoma cases [17]. The following data have been calculated for the individual countries and show a consistent trend: In Italy it is estimated, that 3.8% of melanoma cases are linked to sunbed use, more in women than in men (4.2% vs. 3.1%). The effect is particularly pronounced in young individuals, with sunbeds causing 17% of melanomas in those aged 35 years or younger [19]. For France, a total of 382 melanomas occurring in adults over 30 years in 2015 were likely attributed to the use of sunbeds, equivalent to 1.5% and 4.6% of all melanoma cases in men and women, respectively [20]. In Germany, approximately 4.7% of malignant melanomas in 2018 were attributable to ‘ever’ use of sunbeds. Additional analyses indicated that the vast majority of these cases (3.8%) could be due to highly frequent sunbed use (>10 times/year) [21]. These observations align with the knowledge that indoor tanning is significantly linked to well-established risk factors for melanoma, including a high number of nevi, the presence of atypical nevi, and sun damage. While still a matter of controversy, it seems that sunbeds have a causative role in melanoma [22,23].

Diet emerges as an additional lifestyle risk factor of melanoma. The average lifetime consumption of liquors and spirits showed to be significantly correlated with melanoma, with the highest intake (>3.08 g/day) associated with a 47% increased melanoma risk compared with the lowest intake (0–0.13 g/day). Additional analyses of skin cancer sites suggested that the positive association between alcohol intake and melanoma risk were stronger for tumors occurring on the trunk compared with those of the head, neck or extremities [24,25].

Immunosuppression is a long-known risk factor for melanoma, seen for example in HIV infection, iatrogenic post-organ transplantation immunosuppression, or in the context of lymphoproliferative disorders. The melanoma risk depends on the nature, duration, and intensity of immunosuppression. Notably, HIV-positive Caucasians exhibit a tenfold increase in melanoma incidence rates [26]. Among kidney and heart transplant recipients, the estimated incidence of melanoma is up to fivefold higher. It is known that in both sexes, renal transplant recipients have a 3.6-fold (95% CI: 3.1–4.1) increased rate of melanoma compared with the general population. Male recipients were 3.8 times more likely (95% CI: 3.2–4.4) to develop melanoma compared with men in the general population (*p* = 0.0001), while female recipients were 1.9 times more likely (95% CI: 1.4–2.7) compared with women in the general population. Among African Americans, who have a low melanoma prevalence, the incidence of melanoma in renal transplant recipients was 17.2 times greater than the reported rate for African Americans in the general population (13.32 per 100,000 population vs. 0.776 per 100,000 population, *p* = 0.0001) [27].

Moreover, older age, male gender, Caucasian ethnicity, and the use of ciclosporin and tacrolimus therapies are associated with a substantially increased risk of melanoma in OTR [28,29,30]. The risk for melanoma more than doubles for individuals with non-Hodgkin’s lymphoma (RR: 2.4, 95% CI: 1.8–3.2) or with chronic lymphocytic leukemia (RR: 3.1, 95% CI: 2.1–4.4) [31].

**Table 1:** Extrinsic risk factors for melanoma: Solar and artificial UV radiation, lifestyle habits, and immunosuppression. (Abbreviations: RR = Relative Risk, CI = Confidence Interval, HR = Hazard Ratio, IR = Incidence Rate, *p* < 0.05 for all presented values).

Extrinsic Risk Factors for Melanoma		
Solar UV radiation		[8,11,12]
● Intermittent sun exposure	RR: 1.61 (95% CI: 1.31–1.99)	[9,14]
● Sunburn (especially in childhood)	RR 2.03 (95% CI: 1.73–2.37)	[9,15,16,17]
● Sunbathing (‘ever’ intentional sun exposure)	RR 1.44 (95% CI: 1.18–1.76)	[11,19]
Artificial UV radiation		
● ‘ever’ sunbed use use <35 years old	RR: 1.2 (95% CI: 1.08–1.34) RR: 1.59 (95% CI: 1.36–1.85)	[17,19,20,21]
Lifestyle factors		
● High lifetime intake of spirits	HR: 1.47 (95% CI = 1.08–1.99)	[24,25]
Immunosuppression		
● HIV in Caucasians	IR > 10 fold increased	[26]
● (renal) Transplant recipients	RR: 3.6 (95% CI: 3.1–4.1)	[27,28,29,30]
● Non-Hodgkin’s lymphoma	RR: 2.4 (95% CI: 1.8–3.2)	[31]
● Chronic lymphocytic leukemia	RR: 3.1 (95% CI: 2.1–4.4)	[31]

### 2.2. Intrinsic Risk Factors for Melanoma

Apart from extrinsic and behavioral risk factors associated with melanoma, there are also intrinsic ones. It is estimated that 55% of the variation in liability to melanoma is due to genetic influences [32]. Some intrinsic risk factors can be discerned from the individual’s phenotype. Considering skin color and type, the risk significantly increases with fair skin compared with dark skin. Remarkably, a recognizable dose-response trend emerges, with the Fitzpatrick phototype classification showing a progressive risk pattern: individuals with phototype III have an 70% increased risk for cutaneous melanoma compared with individuals with phototype IV (RR: 1.77, 95% CI: 1.23–2.56). Phototype II compared with IV is linked to an approximately 80% increased risk (RR: 1.84, 95% CI: 1.43–2.36), and the risk for cutaneous melanoma more than doubles when comparing phototype I to IV (RR: 2.09, 95% CI: 1.67–2.58). In addition to tanning ability and the tendency to sunburn, eye color is linked to the risk of developing cutaneous melanoma: having light eye color (blue or green vs. dark) is associated with an approximately 50% increased risk (RR: 1.47, 95% CI: 1.28–1.69 and RR: 1.61, 95% CI: 1.06–2.45, respectively). Hair color further contributes to the risk profile, with red hair carrying more than triple the risk compared with dark hair color (RR: 3.64, 95% CI: 2.56–5.37). Blonde hair poses an almost double risk (RR: 1.96, 95% CI: 1.41–2.74), while light-brown hair is associated with an approximately 60% increased risk compared with dark hair color (RR: 1.62, 95% CI: 1.11–2.34) [33]. Finally, freckles also play a role, as their high-density presence is associated with more than a twofold increased risk (RR: 2.10, 95% CI: 1.80–2.45) [34].

Another well-known risk factor, probably arising from an interplay of external influences, such as sun exposure and genetic predisposition, is the presence of nevi. A systematic meta-analysis confirmed that the number of common nevi and atypical nevi substantially influences the likelihood of cutaneous melanoma. The risk for people with a very high number of nevi (101–120) was found to almost seven times greater (RR: 6.89, 95% CI: 4.63–10.25) than for people with very few nevi (<15) [35]. A high nevus count is thereby strongly associated with melanoma on sites not usually exposed to the sun (*p* < 0.001) [36]. Moreover, the existence of abnormal or irregular nevi is recognized as a risk factor. The presence of 5 atypical nevi increases the risk of melanoma by 6 (RR: 6.36, 95% CI: 3.80–10.33), compared with individuals without any atypical nevus. The etiology of nevi is complex and is probably due to the interaction of multiple genes and environmental factors. Understanding the etiology of nevi and their changes during tumor progression, is important to better understand the pathomechanism of melanoma [35].

Nevus-associated melanomas represent 26–28% [37,38]. Traditionally, progression from nevi to melanoma was seen as linear, but in individual cases, stages may be skipped. Nevi constitute growth arrested, clonal neoplasms of melanocytes, caused by mutations in the mitogen-activated protein kinase (MAPK) pathway, most commonly by BRAFV600E-activating mutation. Most nevi will never progress to melanoma; some remain stable, while others regress. The low overall rate of nevus progression to melanoma suggests that robust tumor-suppressive mechanisms are initiated following BRAF and other mutations [39].

When considering gender-specific perspectives for melanoma, distinct patterns emerge in the incidence and localization of melanoma. Incidence rates among females aged 15–29 years are significantly higher than in males in the same age group (RR: 2.32; *p* < 0.05). Females being more affected in younger years, rates among both sexes increase with age, and differences between them attenuate with older age. From the age of 50 years onwards the incidence of melanoma among men is greater than in women [40,41]. At all ages, mortality rates are higher in males, whereas females show a highly consistent and independent advantage in overall survival, disease-specific survival, time to lymph node metastasis, and time to distant metastasis. These observations suggest differences in tumor–host interaction across gender [42]. In addition, gender-specific differences were found regarding tumor localization. In males, the trunk region predominates, comprising approximately 46.8% of melanoma cases, whereas women develop melanoma most frequently on the hip and lower extremities (39.5%) [43,44]. The observed differences may be linked to gender-specific clothing and sun exposure habits. Different risk patterns are particularly noticeable for intermittently sun-exposed areas. Sex-specific UV radiation exposure, such as men more commonly exposing their bare torso and women exposing their lower extremities, might contribute to melanoma risk in these regions [44,45].

Other intrinsic risk factors may be less apparent, but yet, are crucial: a personal medical history of skin cancer significantly increases the risk for melanoma. Melanoma survivors face an approximately 9-fold increased risk of developing subsequent melanoma compared with the general population. Furthermore, an individual’s personal history of BCC is associated with an increased incidence of melanoma when compared with those without a BCC diagnosis (2.46% vs. 0.37%; *p* < 0.0001), leading to a 6.6-fold higher risk of melanoma in patients with BCC [46,47].

But not only is personal medical history significant: currently, family history is regarded as one of the most important risk factors for cutaneous melanoma [48]. Relatives of cases diagnosed with melanoma are at considerable lifetime risk of the disease, especially if the case was diagnosed at a young age. The relative risk of melanoma nearly doubles (RR: 1.74, 95% CI: 1.41–2.14) in subjects with first-degree relatives with melanoma and increases with the number of affected family members [34]. The cumulative risk of melanoma rises to 6.9% (6.1%) at age 80 in male (female) first-degree relatives of cases, and to 10.8% (9.5%) in relatives of cases diagnosed before age 50 [49]. Approximately 7–15% of all melanoma cases occur in patients with a family history of melanoma. However, this does not necessarily indicate the transmission of a single genetic mutation in those families. In most cases of familial melanoma, shared experiences of sun exposure among family members with susceptible skin types must be considered [50,51].

Familial melanoma is a term used to describe families in which two or more first-degree relatives have been diagnosed with melanoma. It is a genetic condition and research is ongoing on potential melanoma susceptibility genes. To date, several genes have been linked to familial melanoma. CDKN2A, located on chromosome 9p21, is the major known high-risk melanoma susceptibility gene [52]. The largest familial melanoma sample yet available, included 466 families (2137 individuals) with at least three melanoma patients. The study revealed that CDKN2A mutation frequency varies from <25% to >50% in high-risk families, showing striking differences across geographic locations. These findings indicate that the development of tumors in CDKN2A mutation carriers is dependent on interactions between environmental and host factors other than the germline mutation. Familial melanoma cases with germline mutations in CDKN2A have younger ages at onset (40 vs. 50 years), have increased numbers of melanoma cases per family, and are more prone to developing multiple melanomas and other cancers, such as pancreatic cancer, compared with familial cases lacking CDKN2A mutations [53,54]. Mutations in CDKN2A are also detected in sporadic melanoma patients. The CDKN2A mutation frequency for patients without a family history, but with at least two primary melanoma, is around 8.2% [55]. Many other susceptibility genes have been discovered, including CDK4, BAP1, MITF, TERT, ACD, TERF2IP, POT1 and MC1R [56]. The melanocortin-1-receptor (MC1R) gene is the most common low risk susceptibility gene for melanoma, mutated in 70–90% of familial melanoma [57]. The gene is a key regulator of skin pigmentation by stimulating the preferential production of brown-black eumelanin compared with the red-yellow pigment of phaeomelanin [58]. MC1R is highly polymorphic and variants resulting in a partial loss of the receptor’s signaling ability are associated with a quantitative shift of melanin synthesis from eumelanin to phaeomelanin, which is associated with the red hair color (RHC) phenotype [59]. It was demonstrated that individuals carrying a single MC1R variant exhibit 1.41 higher odds (95% CI: 1.07–1.87) of developing sporadic cutaneous melanoma compared with wildtype homozygous subjects. Moreover, carriers with two or more MC1R variants demonstrated 2.51 increased odds (95% CI: 1.83–3.44) of developing cutaneous melanoma. Interestingly, when considering phenotypic characteristics, it was shown that MC1R–associated melanoma risk increased only for darker-pigmented Caucasians, subjects with no freckles, no red hair and skin type III or IV. For individuals with the RHC phenotype, the risk of cutaneous melanoma was not independently predicted by having MC1R variants [60].

Additionally, melanoma risk is elevated in mixed cancer syndromes caused by mutations in PTEN, BRCA1, BRCA2, RB1, BAP1 and TP53 [56]. The BRCA1-associated protein-1 (BAP1) is a tumor suppressor gene, whose somatic or germline mutation predisposes patients or families to cancers including uveal melanoma, mesothelioma, renal cell carcinoma and melanoma. BAP1 tumor predisposition syndrome is inherited in an autosomal dominant pattern, characterized by multiple skin-colored, elevated melanocytic tumors in the individuals concerned [61,62]. The incidence of cutaneous melanoma in carriers of pathogenic BAP1 variants is increased, with varying impacts depending on the type of genetic variant. It was shown, for example, that the median age of melanoma diagnosis is lower in null than missense variant carriers (39 vs. 57 years) [63]. The diagnosis of BAP1-inactivated melanomas represents a clinical and histopathological challenge, requiring comprehensive analysis of morphology and sometimes molecular analysis in addition to immunohistochemistry [64].

In addition to the various mutations and tumor predisposition syndromes considered, it has been demonstrated that the overall susceptibility to melanoma is higher in individuals with a history of a prior malignant disease. There is a notable increase in standardized incidence ratios (SIRs) subsequent to diagnoses of other malignancies, such as breast cancer (SIR: 5.13, 95% CI: 3.91–6.73), thyroid cancer (SIR: 16.2, 95% CI: 5.22–50.2), head and neck cancer (SIR: 5.62, 95% CI: 1.41–22.50), soft tissue cancer (SIR: 8.68, 95% CI: 2.17–34.70), cervical cancer (SIR: 12.5, 95% CI: 3.14–50.20), kidney and urinary tract cancer (SIR: 3.19, 95% CI: 1.52–6.68), prostate cancer (SIR: 4.36, 95% CI: 2.63–7.24) and acute myeloid leukemia (SIR: 6.44, 95% CI: 2.42–17.20). Conversely, melanoma survivors are at an increased overall risk of developing a second primary malignancy (RR: 1.57, 95% CI 1.29–1.90). Elevated standardized incidence ratios (SIR) are observed at specific sites: bone (SIR: 2.09, 95% CI 1.08–4.05), non-melanoma skin cancer (SIR: 4.01, 95% CI 1.81–8.87), soft tissue (SIR: 6.80, 95% CI 1.29–35.98), colon-rectum (SIR: 1.12, 95% CI 1.00–1.25), female breast (SIR: 1.14, 95% CI 1.07–1.22), kidney (SIR: 1.34, 95% CI 1.23–1.45), prostate (SIR: 1.25, 95% CI 1.13–1.37), non-Hodgkin lymphoma (SIR: 1.37, 95% CI 1.22–1.54), and chronic lymphatic leukemia (SIR: 2.74, 95% CI: 2.43–3.08). These observations can probably be attributed to the aggregation of lifestyle risk factors within specific subgroups of the population, primarily influenced by sociocultural factors and surveillance bias [65,66,67].

**Table 2:** Intrinsic risk factors for melanoma: Phenotypic traits, sex, medical history, genetic conditions, and susceptibility genes. (Abbreviations: RR = Relative Risk, CI = Confidence Interval, IR = Incidence Rate, O:E = Observed to Expected ratio, SIR = Standardized Incidence Ratio, OR = Odds Ratio, *p* < 0.05 for all presented values).

Intrinsic Risk Factors for Melanoma		
Phenotype		
● Fitzpatrick phototype:		[33]
III vs. IV	RR: 1.77 (95% CI: 1.23–2.56)	
II vs. IV	RR: 1.84 (95% CI: 1.43–2.36)	
I vs. IV	RR: 2.09 (95% CI: 1.67–2.58	
● Light eye color:		[33]
blue vs. dark	RR: 1.47 (95% CI: 1.28–1.69)	
green vs. dark	RR: 1.61 (95% CI: 1.06–2.45)	
● Light hair color:		[33]
red vs. dark	RR: 3.64 (95% CI: 2.56–5.37)	
blond vs. dark	RR: 1.96 (95% CI: 1.41–2.74)	
light brown vs. dark	RR: 1.62 (95% CI: 1.11–2.34)	
● Freckles	RR: 2.10 (95% CI: 1.80–2.45)	[34]
● High nevi count (>100)	RR: 6.89 (95% CI: 4.63–10.25)	[35]
● Presence of atypical nevi	RR: 6.36 (95% CI: 3.80–10.33)	[35]
Sex		
● Male sex	IR per 100,000: male vs. female 29.3 vs. 18.0	[40,41]
Medical history		
● Personal history of		
melanoma	O:E: 8.61 (95% CI: 8.31–8.92)	[46,47]
BCC (yes vs. no)	2.46% vs. 0.37%	[46]
● Family history of melanoma	RR: 1.74 (95% CI: 1.41–2.14)	[34,48,50]
● Preceding malignancy:		[65,66]
Breast cancer	SIR: 5.13 (95% CI: 3.91–6.73)	
Thyroid cancer	SIR: 16.2 (95% CI: 5.22–50.2)	
Head and neck cancer	SIR: 5.62 (95% CI: 1.41–22.50)	
Soft tissue cancer	SIR: 8.68 (95% CI: 2.17–34.70)	
Cervical cancer	SIR: 12.5 (95% CI: 3.14–50.20)	
Kidney/urinary tract cancer	SIR: 3.19 (95% CI: 1.52–6.68)	
Prostate cancer	SIR: 4.36 (95% CI: 2.63–7.24)	
Acute myeloid leukemia	SIR: 6.44 (95% CI: 2.42–17.20)	
Chronic lymphatic leukemia	SIR: 2.74 (95% CI, 2.43–3.08)	[67]
Genetic conditions and susceptibility genes		
● Familial melanoma		[52,53,54]
● CDKN2A, CDK4, BAP1, MITF, TERT, ACD, TERF2IP, POT1 mutation		[56,57,59,60]
● MC1R		
one variant:	OR: 1.41 (95% CI: 1.07–1.87)	
≥two variants	OR: 2.51 (95% CI: 1.83–3.44)	
Mixed cancer syndromes		
● PTEN, BRCA1, BRCA2, RB1, BAP1, TP53 mutation		[56,61,62,63]

## 3. Risk Factors for Basal Cell Carcinoma

Basal cell carcinoma (BCC) is the most common skin cancer worldwide, accounting for over 85% of all NMSCs in Europe [68,69,70]. There are several risk factors that are associated with BCC and, as for melanoma, the development of BCC is an interplay of extrinsic and intrinsic risk factors.

### 3.1. Extrinsic Risk Factors for BCC

Exposure to solar UV radiation is the major external risk factor for the development of BCC (Table 3). In Australia, for example, it is assumed that nearly all BCCs are attributable to high UV exposure [12]. Both, intermittent and chronic sun exposure increase the risk for BCC. Intermittent sun exposure (>7000 h spent at the beach during holidays in a lifetime) is associated with a 2.1 increased odds (95% CI: 1.09–3.95) for BCC [71]. Sunburn often serves as a measure of intermittent sunlight exposure, and it is another, independent, extrinsic risk factor for BCC. Experiencing any sunburn during childhood or later in life is associated with a 1.43 (95% CI: 1.19–1.72) and 1.40 (95% CI: 1.02, 1.45) higher odds, respectively. A dose-dependent effect is observed: the risk of developing BCC doubles every 5 sunburns, regardless of whether they are experienced in childhood or later in life [72]. In addition to acute sun exposure, chronic UV exposure also contributes to the development of BCC. Individuals working outside have an odds ratio (OR) of 2.08 (95% CI: 1.24–3.50) to develop BCC in commonly UV-exposed body sites compared with controls without occupational sun exposure. The risk for outdoor workers increases independently of histological subtype, tumor localization and Fitzpatrick phototype [73]. While both intermittent and chronic sun exposure increase the risk of BCC, the exposure pattern and anatomical location appear to influence the histological subtype: nodular and infiltrative BCC primarily occur on the face, whereas superficial BCCs tend to develop on the trunk. This observation suggests that the nodular subtype is associated with chronic sun exposure, while intermittent sun exposure could be an etiological factor for superficial BCC [69]. This theory is further supported by divergent oncogenic mutations in the BCC subtypes, caused by different types of sun exposure: a significant association was observed between the superficial type of BCC and mutation of the Patched 1 (PTCH1) gene, which in turn was significantly associated with intermittent sun exposure [74]. The correlation between histologic subtype and the body region in which it occurs is further explained by variations in the extracellular matrix, which leads to a different susceptibility of different body regions to the development of specific histological subtypes of BCCs: a recent study highlighted that not all epidermal cells across the body are equally sensitive to oncogenic transformation and stressed the role of the extracellular matrix in the development of BCC. It showed that the composition of the extracellular matrix regulates how susceptible different regions of the body are to tumor initiation and invasion. Whereas the ear epidermis is susceptible to oncogenic transformation, the back skin proved to be profoundly resistant. More precisely, increased stiffness and a denser collagen I network e.g., in the back skin, provides natural resistance against dermal invasion and tumor formation of BCC. Reducing collagen I expression in the back skin overcomes this natural resistance to tumor initiation [75].

In addition to natural UV radiation, the use of artificial radiation sources also poses a risk. Ever exposure to indoor tanning devices increases the risk of BCC by 29 % (RR: 1.29, 95% CI: 1.08–1.53). Furthermore, the age at first exposure is crucial: Indoor tanning before the age of 25 is associated with a 40 % increased risk of BCC (RR: 1.40, 95% CI: 1.29–1.52) [76]. Sunbed use not only increases the overall risk of developing BCC but also contributes to its occurrence at a young age, particularly before 40 years, which is known as early-onset BCC. Indoor tanning has been associated with a 69% increased risk of early onset BCC (95% CI: 1.15–2.48). Through population attributable risk calculations, it is estimated that around 27% of early-onset BCC cases could be averted by abstaining from indoor tanning. Particularly among women under the age of 40, the proportion of preventable cases is even more significant, with 43% of BCCs potentially avoided if females refrained from indoor tanning [77]. This gender-specific observation can be attributed to the higher frequency of tanning bed visits among women across Europe compared with men [78,79].

In addition to intentional, self-selected sources of radiation, medical treatment options also come with inherent side effects. Radiotherapy is a long-known risk factor for BCC. Exposure to radiation during childhood is linked to a substantial 6.3-fold risk (95% CI: 3.5–11.3) of developing NMSC, predominantly BCC (97%). Most of these tumors (90%) occurred within the radiotherapy field. The OR for subjects diagnosed with cancer requiring radiotherapy ≤21 years or younger, and receiving ≥35 Gray or more to the skin site compared with no radiation therapy was 39.8 (95% CI: 8.6–185) [80,81,82]. Not only patients, but also medical staff, are exposed to potentially harmful radiation, particularly those who worked in the more distant past. The relative risk of BCC is elevated in technicians who began their work in the 1950s (RR: 1.42, 95% CI: 1.12–1.80). The risk further increases, when work was initiated in the 1940s (RR: 2.04, 95% CI: 1.44–2.88), and even doubles when the occupation started before 1940 (RR: 2.16; 95% CI: 1.14–4.09), an era characterized by high ionizing radiation exposure, compared with those who commenced work after 1960 [83].

Individual lifestyle also has an influence on the development of BCC. Alcohol consumption, for example, is a risk factor for cutaneous BCC in both women and men. Regarding the different alcoholic beverages, wine and spirits were shown to be significantly associated with BCC development [25,84,85]. One possible explanation is that alcohol consumption has been associated with a higher number and severity of sunburns, indicating that the correlation is primarily due to more risky sun exposure habits [86,87]. Furthermore, it has been hypothesized that the combination of alcohol consumption and UV radiation can potentiate the skin carcinogenicity through the intermediate byproducts or metabolites of alcohol (e.g., acetaldehyde), which can serve as photosensitizers [88].

Conversely, certain lifestyle factors have a protective effect against the development of BCC. Various studies have shown that a high body mass index (BMI) is inversely associated with BCC risk. The substantial reduction in risk associated with a high BMI is noteworthy, as individuals with a BMI over 35 have up to a fourfold lower risk of developing BCC [89,90]. An explanation for this might be a different UV exposure behavior. Individuals with a greater body weight may be less inclined to expose their bodies to UV light in public places and tend to use solariums less than slim people [91].

A significant association between BCC and diseases related to iatrogenic or non-iatrogenic immunosuppression has been observed, for example in the context of organ transplantation or autoimmune diseases [92]. In comparison with the general population, the relative risk of BCC is increased sixfold in OTRs (standardized incidence ratio (SIR): 6.1, 95% CI: 5.4–6.9). The risk is higher in kidney and heart/lung recipients compared with liver recipients (SIR kidney 7.2, 95% CI: 6.3–8.3; SIR heart/lung 5.8, 95% CI: 4.0–8.2; SIR liver 2.6, 1.7–4.0), and it increases over time since transplantation [93].

Furthermore, the use of immunosuppressives, such as methotrexate, is associated with an increased risk of BCC (OR: 1.29, 95% CI: 1.20–1.38), with a linear dose–response effect [94].

**Table 3:** Extrinsic risk factors for BCC: Solar and artificial UV radiation, lifestyle habits, and immunosuppression. (Abbreviations: OR = Odds Ratio, CI = Confidence Interval, RR = Relative Risk, HR = Hazard Ratio, SIR = Standardized Incidence Ratio, *p* < 0.05 for all presented values).

Extrinsic Risk Factors for BCC		
Solar UV radiation		[12]
● Intermittent exposure	OR: 2.1 (95% CI: 1.09–3.95)	[71]
● Chronic exposure/ outdoor workers	OR: 2.08 (95% CI: 1.24–3.50)	[73]
● Sunburn	OR: 1.40 (95% CI: 1.02–1.45)	[72]
during childhood	OR: 1.43 (95% CI: 1.19–1.72)	
Artificial UV radiation		
● Sunbed		[76,77]
‘ever’ use	RR: 1.29 (95% CI: 1.08–1.53)	
use <25 years old	RR: 1.40 (95% CI: 1.29–1.52)	
● Medical radiation		[80,81,82,83]
radiotherapy during childhood	RR: 6.3 (95% CI: 3.5–11.3)	
medical technicians		
start of work in 1950s	RR: 1.42 (95% CI: 1.12–1.80)	
start of work in 1940s	RR: 2.04 (95% CI: 1.44–2.88)	
start of work <1940	RR: 2.16 (95% CI: 1.14–4.09)	
Lifestyle factors		
● Baseline alcohol consumption > 15 vs. 0.1–4.9 g/day	HR: 1.12 (95% CI = 1.01–1.23)	[25,84,85]
Immunosuppression		
● Organ transplant recipients	SIR: 6.1 (95% CI: 5.4–6.9)	[92,93]
● Autoimmune diseases		[92]
Rheumatoid arthritis	OR 1.20 (1.11–1.29)	
● Methotrexate	OR 1.29 (95% CI: 1.20–1.38)	[94]

### 3.2. Intrinsic Risk Factors for BCC

Apart from external factors, inherited genetic susceptibility also contributes to the risk of BCC development (Table 4). Several phenotypic characteristics, in particular light complexion or low Fitzpatrick phototypes, modulate susceptibility to BCC: phototypes I, II and III have been shown to be independent risk factors for the development of BCC compared with phototypes IV–VI. A linear risk progression can be seen, with corresponding ORs of 17.5 (95% CI: 3.29–113.7), 15.6 (95% CI: 7.5–34.3) and 10.4 (95% CI: 5.1–22.4) for phototypes I, II and III, respectively [95]. The ORs of BCC is significantly increased in individuals with blonde (OR: 2.2, 95% CI: 1.27–3.91)) and light blonde to red hair (OR: 2.3, 95% CI: 1.15–4.49). Beyond hair color, the importance of eye color is noteworthy. Having light blue eyes show 1.8 increased odds for BCC (95% CI: 0.94–3.66), with green eyes even associated with a 3.4-fold increase in the odds (95% CI: 1.92–6.22) [71]. Another phenotypic risk factor for BCC are freckles in childhood (OR: 1.57, 95% CI: 1.29–1.92) [96,97].

Furthermore, genome-wide association studies have identified over 30 loci associated with BCC susceptibility [98]. Individuals carrying any MC1R variant have a significantly increased risk of BCC compared with subjects without MC1R variant. Carrying at least one MC1R variant is significantly associated with BCC (OR: 1.48, 95% CI: 1.24–1.76). Carriers of two or more MC1R variants have an even higher chance for developing BCC (OR: 1.70, 95% CI: 1.36–2.12) compared with carriers of one MC1R variant. It is important to notice that the MC1R-associated BCC risk was observed only for subjects without red hair, while in subjects with red hair, MC1R seemed not to have an effect in addition to phenotype. MC1R may therefore contribute to skin tumorigenesis through mechanisms distinct from pigmentation [99].

Other gene mutations associated with BCC are PTCH1, PTCH2, SUFU, and Smoothed (SMO). Especially PTCH1, but also PTCH2 and SUFU pathogenic variants, are associated with the hereditary or sporadic basal cell nevus syndrome (Gorlin-Goltz syndrome), which is characterized by numerous BCC, along with skeletal, ophthalmologic, and neurologic abnormalities. In this particular patient group age, number of sunburns and a history of radiation exposure are significantly associated with the severity of lifetime BCC [100,101].

Previous NMSC increases the risk of developing BCC. The 3-year cumulative risk for developing a subsequent BCC after the first one varies between studies, with a mean of 44% (33–70%). This represents an at least 10-fold increase in incidence compared with the rate in a comparable general population. The risk of developing BCC in patients with a prior cutaneous squamous cell carcinoma (cSCC) is approximately equal to the risk observed among individuals with a prior BCC [102].

For BCC development, gender-specific differences can be observed: male sex is an inherent risk factor for BCC. Being more frequent among females until the age of 40, it preferentially affects older males (>60 years old), leading to men having a higher aggregate risk than women. Age and male sex are two main, nonmodifiable, constitutive risk factors of BCC susceptibility [92,103]. Furthermore, there is a statistically significant association between site and sex. BCCs of the face, lower limb, lip and eyelid were more common in female patients, whereas BCCs of the ear/external auditory canal, scalp/neck, trunk, and upper limb were more common in male patients [104].

**Table 4:** Intrinsic risk factors for BCC: Phenotypic traits, medical history, genetic conditions, sex, and age. (Abbreviations: OR = Odds Ratio, CI = Confidence Interval, RR = Relative Risk, NMSC = Non-Melanoma Skin Cancer, *p* < 0.05 for all presented values).

Intrinsic Risk Factors for BCC		
Phenotype		
● Fitzpatrick phototype		[95]
I vs. IV–VI	OR 17.5 (95% CI: 3.29–113.7)	
II vs. IV–VI	OR 15.6 (95% CI: 7.5–34.3)	
III vs. IV–VI	OR 10.4 (95% CI: 5.1–22.4)	
● Light hair color		[71]
blonde	OR: 2.2 (95% CI: 1.27–3.91)	
Light blonde to red	OR: 2.3 (95% CI: 1.15–4.49)	
● Light eye color		[71]
Light blue	OR: 1.8 (95% CI: 0.94–3.66)	
green	OR: 3.4 (95% CI: 1.92–6.22)	
● Freckles in childhood	OR: 1.57 (95% CI: 1.29–1.92)	[96,97]
Medical history		
● Previous NMSC	3-year risk: 44% (33–70%)	[102]
Genetic conditions & susceptibility genes		
● PTCH1, PTCH2, SUFU, SMO		[100,101]
one MC1R variant	OR: 1.48 (95% CI: 1.24–1.76)	
≥two MC1R variants	OR: 1.70 (95% CI: 1.36–2.12)	
Sex		
● Male sex	RR: 1.27 (95% CI: 1.25–1.29)	[92,103,104]
Age		[92]

## 4. Risk Factors for Cutaneous Squamous Cell Carcinoma

cSCC globally ranks as the second most common form of skin cancer after BCC [70]. As the latter, it develops through an interplay of external (Table 5) and intrinsic (Table 6) risk factors.

### 4.1. Extrinsic Risk Factors for SCC

Chronic sun exposure is directly correlated with cSCC and considered a major environmental risk factor [105]. According to previous studies, almost 85% of cSCC in white populations worldwide are attributable to excessive UV radiation [106]. The incidence and risk depend on the geographical location. For Australia, it is estimated that nearly all cSCC are attributable to the high UV exposure, whereas in Canada it is about 83% of cSCCs [12,107]. Individuals who work outdoors are chronically sun-exposed, and occupational UV-exposure has been shown to be a major risk factor for cSCC [108]. Workers with intense exposure (>3878 weighted hours of exposure in a lifetime) have a significantly increased risk of cSCC, presenting an OR of 2.2 (95% CI: 1.13–4.08). A linear dose-response relationship between occupational UV-exposure and incident cSCC is observed [71,108]. This has led to the recognition of cSCC as an occupational disease for outdoor workers in several countries, like Denmark, France, Romania, Italy, Portugal, Czech Republic and Germany [109,110]. Consequently, cSCC tends to develop preferentially on chronically sun-exposed areas of the skin: approximately 70% of all cSCC develop on the face and head. Furthermore, tumors tend to be more aggressive, with significantly more high-risk subtypes developing in these sites [69,108]. A characteristic mutational signature caused by UVB-irradiation, with prevalent C→T transitions, is found in cutaneous cSCC. TP53 mutations are already prevalent in actinic keratosis (AK) and in aggressive or metastatic cSCC, the mutation rate can reach 79–95% [111,112,113]. In addition to chronic and cumulative sun exposure, intermittent sun exposure, particularly manifested through sunburns, constitutes a risk factor. A history of blistering sunburn is significantly associated with cSCC (OR: 2.02, 95% CI: 1.22–3.33) [114].

In addition to natural UV radiation, the use of artificial radiation sources such as tanning beds is associated with cSCC development: performing indoor tanning conveys a 58% increased risk for cSCC (RR: 1.58, 95% CI: 1.38–1.81). Concerning the type of indoor tanning devices, both sunlamp (RR: 1.72, 95% CI: 1.16–2.53) and sunbed use (RR: 1.48, 95% CI: 1.20–1.83) are risk factors for cSCC. First exposure at a young age is particularly dangerous: using indoor tanning devices before the age of 20 nearly doubles the risk for cSCC (RR: 1.89, 95% CI: 0.90–3.98) [115]. Similarly, the excessive use of tanning beds (>240 sessions), as observed in a Norwegian study on women, is associated with a nearly twofold increased risk of developing cSCC (RR: 1.83, 95% CI: 1.38–2.42) [116].

Immunosuppression is a medical condition strongly linked to cSCC development. Immunocompromised patients are significantly younger at the time of cSCC diagnosis, compared with non-immunocompromised cSCC patients (68 vs. 78 years), display higher recurrence rates (24% vs. 15%) and have more often multiple cSCCs (28% vs. 10%) [117]. The association between immunosuppression and cSCC risk was reported for several underlying conditions like chronic lymphocytic leukemia (4.82 fold increased cSCC risk) or HIV (5.40 fold increased cSCC risk), but it is especially strong for solid organ transplant recipients (OTR) [67,118]. Indeed, in immunocompromised solid OTRs, the incidence of cSCC is approximately 100 times greater than the general population [119]. CSCC in OTR show a higher regulatory T cell/cytotoxic T cell ratio, believed to favor immune evasion and progression of the tumor [120]. Therefore, regulatory T cells are proposed to predict the risk of cSCC after transplantation [121]. Risk factors for posttransplant skin cancer include pretransplant skin cancer (HR: 4.69, 95% CI: 3.26–6.73), male sex (HR: 1.56, 95% CI: 1.34–1.81), thoracic organ transplantation (HR: 1.51, 95% CI: 1.26–1.82), age at transplant ≥ 50 years (HR: 2.77, 95% CI: 2.20–3.48) and white ethnicity (HR: 9.04, 95% CI: 6.20–13.18) [122]. The risk of cSCC after organ transplantation increases linearly with each incremental decrease in Fitzpatrick skin type, from VI to I [123]. The increased risk of cSCC in OTR undergoing immunosuppressive treatment is linked to various pathogenetic mechanisms. These mechanisms involve not only the weakening of the immune system, and thus decreased ability to protect against UV radiation, viral infection, and DNA damage, but also direct impact like photosensitization, such as in the case of azathioprine: through the accumulation of 6-thioguanine in patients’ DNA, UVA-mediated oxidative stress and mutagenic DNA lesions are caused [124]. The risk of cSCC in OTRs treated with azathioprine is significantly increased by 56% compared with those not treated with azathioprine but with other immunosuppressant drugs (RR: 1.56, 95% CI: 1.11–2.18) [125].

The association between the greatly increased incidence of cutaneous cSCC in OTR and a possible viral etiology, specifically β human papillomaviruses (HPV), has been intensely investigated over the years.

In a broad meta-analysis it was shown that cSCC from immunosuppressed patients are more likely to carry HPV than cSCC from immunocompetent patients (pooled ES: 3.01, 95% CI: 2.00–4.52) [126].

In both immunocompromised and non-immunosuppressed individuals HPV was shown to be involved in the pathogenesis of cSCC: cSCCs are more commonly positive for HPV DNA than healthy skin samples (OR: 2.13, 95% CI: 1.13–4.03). Furthermore, the presence of HPV DNA was found in skin sites classified as having been extensively exposed to the sun, both in lesions and in healthy skin [127]. The preferential finding of HPV DNA in sun-exposed skin sites might be related to local UV-immunosuppressive effects [128].

Moreover, the risk of cSCC is impacted by individual lifestyle factors: a broad meta-analysis revealed an inverse relationship between body mass index (BMI) and cSCC development (RR: 0.88, 95% CI: 0.85–0.91) [129], possibly explained by the same considerations made for BCC. Alcohol appears to have the opposite effect, with a positive, linear association between alcohol intake and cSCC: baseline alcohol intake of more than 15 g/day was positively associated with cSCC compared with an intake of 0.1–4.9 g/day (HR: 1.44, 95% CI: 1.17–1.77) [25].

Chronic skin lesions or infections, as well as skin inflammation from burns, scars, and other conditions, represent another potential origin for cSCC. Indeed, cSCC is the most common neoplasm on burn scars and occurs after a mean latency period of 30 years in 50-year-old individuals after a burn injury. In this context, the major risk factors for post-burn cSCC development are healing by secondary intention, non-healing wounds, and fragile scars that ulcerated and were easily traumatized [130].

**Table 5:** Extrinsic risk factors for SCC: Solar and artificial UV radiation, immunosuppression, viral infections, lifestyle habits, and chronic skin lesions. (Abbreviations: OR = Odds Ratio, CI = Confidence Interval, RR = Relative Risk, HR = Hazard Ratio, SIR = Standardized Incidence Ratio, HPV = Human Papillomavirus, OTR = Organ Transplant Recipients, *p* < 0.05 for all presented values).

Extrinsic Risk Factors for SCC		
Solar UV radiation		
● Chronic exposure/ outdoor workers	OR: 2.2 (95% CI: 1.13–4.08)	[71,105,108]
● Sunburn	OR: 2.02 (95% CI: 1.22–3.33)	[114]
Artificial UV radiation		
● Sunbed use		[115,116]
‘ever’	RR: 1.48 (95% CI: 1.20–1.83)	
use <20 years old	RR: 1.89 (95% CI: 0.90–3.98)	
excessive use (>240 sessions)	RR: 1.83, 95% CI: 1.38–2.42	
Immunosuppression		
● Organ transplant recipients		[119]
● Azathioprine treatment in OTR	RR: 1.56 (95% CI: 1.11–2.18)	[124,125]
● HIV	SIR: 5.40 (95% CI: 3.07–9.52)	[118]
● Chronic lymphocytic leukemia	SIR: 4.82 (95% CI: 4.57–5.07)	[67]
Viral infections		
● HPV DNA in cSCC	OR: 2.13 (95% CI: 1.13–4.03)	[126,127]
Lifestyle factors		
● Baseline alcohol consumption > 15 vs. 0.1–4.9 g/day	HR: 1.44 (95% CI: 1.17–1.77)	[25]
Chronic skin lesions		
● Burns/scars		[130]

### 4.2. Intrinsic Risk Factors for SCC

In addition to the external factors contributing to the development of cSCC, inherited genetic susceptibility also adds to the risk. Phenotypic characteristics significantly associated with the development of cSCC are especially light pigmentary traits. Individuals with blue or light eye colors have a 19% higher risk of cSCC (RR: 1.19, 95% CI: 1.01–1.41), while those with hazel, green, or medium eye colors face a 24% higher risk (RR: 1.24, 95% CI: 1.06–1.45) compared with those with dark or brown eye color [131]. Having blonde hair was also shown to be an independent risk factor (OR: 2.4, 95% CI: 1.26–4.64). Furthermore, people whose skin becomes red and does not tan also show a significant association with cSCC development (OR: 2.7, 95% CI: 1.67–4.27) [71].

Over 60 susceptibility loci for cSCC have been detected by recent multi-trait genetic analysis and show high genetic correlation with BCC, melanoma, pigmentation traits, autoimmune diseases, and blood biochemistry biomarkers. These include ATM, DSTYK, GPR98, and SOX6 [132]. Carrying at least one MC1R variant increases the risk of cSCC (OR: 1.61, 95% CI: 1.35–1.91). Carriers of two or more MC1R variants present an even higher OR compared with subjects carrying one MC1R variant (OR: 2.10, 95% CI: 1.60–2.76) [99].

Furthermore, gender-specific differences can be observed. Male sex is an inherent risk factor for cSCC. Several studies suggest that age-standardized incidence rates in males are twice as high as in females [133,134]. This risk is especially pronounced in cutaneous cSCC of the head and neck (HNcSCC). Overall, more than 70 % of HNcSCC affect men (71%, CI: 67–74). Males are significantly more affected by cSCC of the ear (92%, CI: 89–94), lip (74%, CI: 66–81), and eyelid (56%, CI: 51–62) [135]. Furthermore, male gender is associated with an approximately 1.7 times higher risk of developing metastases [134,136].

Once diagnosed with a cSCC, the risk of a second one increases: the 3-year cumulative risk of a subsequent cSCC after an index cSCC is 18%, at least a 10-fold increase in incidence compared with the incidence of first tumors in a comparable general population. The risk of developing an cSCC after having had a BCC is significantly lower, with a cumulative 3-year mean of 6% [102]. Another lesion associated with cSCC development is AK. Approximately 10 % of AK advance to cSCC, and the progression will take approximately 2 years (24.6 months (95% CI: 21.04–28.16 months) [137,138]. This leads to another important risk factor for cSCC, which is age: cSCCs primarily occur in individuals aged 60 and above, with the incidence significantly increasing with age [139,140]. Over the past 30 years, the incidence of cSCC increased particularly in the older, and is expected to continue to do so. Between 1990 and 2020, the highest increase occurred in the age groups ≥ 60 years, especially in men aged ≥ 80 years, with a three to five-fold increase. Extrapolations up to 2044 showed an unrestrained increase in incidence rates in all countries investigated [141]. This trend will have a significant impact on the current and future burden on dermatologic healthcare, posing major challenges. Therefore, it becomes increasingly important reliably to identify individuals at particularly high risk, enabling them to undergo targeted early detection and therapy. This is pursued through the utilization of risk scores.

**Table 6:** Intrinsic risk factors for SCC: Phenotypic traits, medical history and conditions, genetic conditions, sex, and age. (Abbreviations: OR = Odds Ratio, CI = Confidence Interval, RR = Relative Risk, IR = Incidence Rate, *p* < 0.05 for all presented values).

Intrinsic Risk Factors for SCC		
Phenotype		
● Eye color		[131]
Light/blue vs. dark	RR: 1.19 (95% CI: 1.01–1.41)	
Hazel/green/medium vs. dark	RR: 1.24 (95% CI: 1.06–1.45)	
● Blonde hair	OR: 2.4 (95% CI: 1.26–4.64)	[71]
● Inability to tan	OR: 2.7 (95% CI: 1.67–4.27)	[71]
Medical history & conditions		
● Previous SCC	3-year risk: 18% (9–23%)	[102]
● Previous BCC	3-year risk: 6% (1–19%)	
● Presence of actinic keratosis		[137,138]
Genetic conditions & susceptibility genes		
● ATM, DSTYK, GPR98, SOX6		[99,132]
one MC1R variant	OR: 1.61 (95% CI: 1.35–1.91)	
≥two MC1R variants	OR: 2.10 (95% CI: 1.60–2.76)	
Sex		
● Male sex	IR: 207.5 (95% CI: 193.9–221.1) per 100,000 persons vs. 128.8 (95% CI: 119.4–138.2) in females	[133,134,136]
Age		[139,140]

## 5. Risk Assessment Innovations and Personalized Medicine in Skin Cancer

As our understanding of cancer and its associated risk factors expands with clinical and experimental evidence, the goal is to formulate advanced strategies for proactive prevention, early detection, and effective treatment. Many strategies for early detection are available such as the widespread practice of screening, but overdiagnosis and overtreatment are prevalent with this approach. Recent research has thus been focused on the development of improved, precise models of cancer prevention and early detection. The primary objective of these tools is to identify high-risk individuals due to known genetic, behavioral, and environmental factors. Optimizing the implementation of detection programs, establishing personalized monitoring plans, and anticipating appropriate treatment modalities and intensities is the ultimate goal [5].

Regarding skin cancer, screening asymptomatic individuals with a full-body examination has not provided enough evidence to demonstrate its effectiveness in reducing morbidity and mortality [4]. Overdiagnosis and overtreatment of skin cancer are common, leading to psychological, economical, and medical consequences [142]. Thus, adapting screening recommendations to the individual risk of patients could be a more (cost)-effective approach [6]. Risk prediction models aim to offer a comprehensive analysis, considering factors such as genetic predisposition, environmental influences, and clinical characteristics, to determine the risk of skin cancer more accurately. Various approaches involve harnessing extensive datasets to construct models that facilitate early detection. Regrettably, these models often lack sufficient validation and inclusion of all known risk factors [7]. A recently introduced risk stratification model attempts to overcome these problems. The authors from 23andMe developed a validated disease risk score based on a questionnaire and genetic data, which could be used in early screening programs. By combining 31 risk factors, a risk score (DRSA) was developed. This age independent disease score predicts the occurrence of skin cancers (BCC, SCC, melanoma) in individuals aged 30 years or older. Participants in the top percentile were not only diagnosed on average 10–14 years earlier than the participants with average scores, but also presented more severe and recurrent forms of skin cancer. The established score and the lifetime risk trajectories could be used in early detection programs to identify asymptomatic individuals with high risk of developing skin cancer, and to predict when they are likely to develop the disease [89].

A particular patient group is represented by OTR. As already mentioned above, NMSC is the most common malignancy in solid organ transplant recipients and a significant cause of morbidity and mortality [143,144]. Therefore, there is a need to develop risk scores that identify particularly vulnerable individuals. The Skin and UV Neoplasia Transplant Risk Assessment Calculator (SUNTRAC) tool has been developed in the US to facilitate the identification of solid organ transplant recipients at a higher risk of skin cancer by stratifying patients into risk groups. Risk factors (white race, pretransplant history of skin cancer, age ≥ 50 years, male sex, thoracic transplant) were assigned weighted point values, resulting in a 4-tier system. The 5-year cumulative incidence of development of skin cancer was 1.01%, 6.15%, 15.14%, and 44.75%, for low, medium, high, and very high SUNTRAC categories, respectively. The tool has been externally validated and tested for applicability in European populations, with similar good prognostic discrimination results. This model could help prioritize and provide better screening and surveillance for these patients, as well as to define screening guidelines for OTR [145,146,147]. Furthermore, evaluation of the clinical utility of these tools in identifying individuals with an elevated risk of skin cancer before specific clinical examinations are undertaken is ongoing.

Another important aspect of risk scores for skin cancer is their role in promoting public awareness of the disease. By encouraging people to know their individual risk and take appropriate measures, risk scores can contribute to raising awareness of the importance of prevention and early detection. This can be achieved through targeted awareness campaigns, training programs for healthcare providers, and public health initiatives [148].

There has been recent growing interest in the involvement of non-coding RNAs (ncRNAs) in the pathogenesis of cancers. Evidence suggests that ncRNAs regulate key tumor pathways and are involved in almost all human tumors, including skin cancer. These functional RNA molecules lack any protein-coding activity, but operate at transcriptional, post-transcriptional, and epigenetic levels and are involved in cancer cell proliferation, angiogenesis, invasion and metastasis [149,150]. Certain ncRNAs can serve as biomarkers for cancer as their expression correlates with specific cancer types or disease progression [151]. Evidence has demonstrated that ncRNAs play crucial roles for the early diagnosis, prognosis, and treatment of melanoma [152,153]. A recent meta-analysis found a combined sensitivity of long ncRNAs in diagnosing melanoma to be 72.4%, with a pooled specificity of 81.2% and an overall area under the curve (AUC) of 0.837. For prognostic approaches, the HR for overall survival, progression-free survival, and disease-free survival were 2.723 (95% CI: 2.259–3.283), 2.913 (95% CI: 2.050–4.138), and 2.760 (95% CI: 2.009–3.792), respectively [153]. These findings indicate that ncRNAs could serve as innovative diagnostic and prognostic biomarkers, potentially enhancing patient management in the future. The possible analysis of alterations in ncRNA profiles, combined with computer-assisted predictions of mRNA targets, facilitates the research into aberrant molecular pathways in cancer cells. This understanding offers valuable insights into tumor responses to various treatment modalities, like drug therapies, radiotherapy, and chemotherapy. Moreover, it helps to estimate the tumor’s resistance to apoptosis and its capacity to evade immune surveillance. By employing a comprehensive approach integrating disciplines such as molecular biology, biochemistry, high-throughput sequencing, and artificial intelligence-driven data analysis, personalized medicine emerges as a viable option for clinical implementation [151].

The expansion of precision medicine in the next decade will be focused on three key areas: complex algorithms, health apps, and ‘omics’-based tests. These advances may cause significant potential benefits for patients, particularly with the anticipated reduction in ‘omics’ testing costs and the enhanced efficiency for manufacturers in developing targeted therapies. However, the adoption of these innovations necessitates adaptation within health technology assessment and guideline-producing agencies. The rapid pace of technological discovery, coupled with the potential complexity and uncertainty of treatment pathways, presents new challenges that must be addressed [154].

Personalized medicine, aimed at tailoring therapeutic approaches to individual genetic profiles, introduces ethical dilemmas for decision-makers. These encompass a wide range of concerns, from safeguarding individual privacy and preventing discrimination based on ethnicity to ensuring equal access and fair allocation of resources. Establishing standards is crucial to ensure equitable treatment for all individuals [155].

Moreover, there is a risk that guidelines may have a shorter lifespan and structural uncertainties may increase. The impracticality of vague recommendations has been highlighted and an urgently needed call for clarity has been made. Clinicians who must make realistic decisions in an uncertain environment require action-oriented guidance [156]. On the other hand, the use (and misuse) of clinical guidelines in medical liability litigation must not be overlooked: clinical guidelines can serve as evidence in court and represent medical expertise influencing the legal standard of care. Although various initiatives have attempted to limit the use of guidelines in legal proceedings, experiences show that their role in litigation can have serious ramifications [157].

## 6. Conclusions

In conclusion, as our understanding of skin cancer and its associated risk factors evolves, the aim is to develop advanced strategies for proactive prevention, early detection, and effective treatment. While conventional screening practices have encountered challenges such as overdiagnosis and overtreatment, recent research projects focus on refining models for cancer screening and early detection. Personalized risk prediction models, exemplified by the 23andMe’s Disease Risk Score, the SUNTRAC tool for organ transplant recipients or the use of ncRNA prediction models, show promising potential for improving screening effectiveness. These models aim to identify high-risk individuals, prioritize screening resources, and enhance surveillance protocols. The expansion of personalized medicine holds significant potential benefits for patients but requires adaptation within health technology assessment and guideline-producing agencies due to new challenges arising from technological advancements and ethical dilemmas.

## Data Availability

Data derived from public domain resources.
